# Cyr61 participates in the pathogenesis of acute lymphoblastic leukemia by enhancing cellular survival via the AKT/NF-κB signaling pathway

**DOI:** 10.1038/srep34018

**Published:** 2016-10-11

**Authors:** Xianjin Zhu, Yanfang Song, Conglian Wu, Chuxi Pan, Pingxia Lu, Meihua Wang, Peizheng Zheng, Rongfen Huo, Chenqing Zhang, Wanting Li, Yulin Lin, Yingping Cao, Ningli Li

**Affiliations:** 1 Fujian Medical University Union Hospital, 29 Xinquan Road, Fuzhou 350001, China; 2 Affiliated Renmin Hospital of Fujian University of Traditional Chinese Medicine, 602 Bayiqi Road, Fuzhou 350001, China; 3 University of Toronto, 27 King’s College Circle, Toronto M5S1A1, Canada; 4 Shanghai Institute of Immunology, Institute of medical sciences, Shanghai Jiao Tong University School of Medicine, 280 South Chongqing Road, Shanghai 200025, China; 5 Fujian Medical University, 88 Jiaotong Road, Fuzhou 350001, China

## Abstract

Cyr61 (CCN1) is the product of a growth factor–inducible immediate early gene and is involved in cell adhesion, survival, proliferation, and differentiation. Cyr61 is overexpressed in human tumors and is involved in the development of tumors. However, the role that Cyr61 plays in acute lymphoblastic leukemia (ALL) cells remains undetermined. The aim of this study was to identify the role of Cyr61 in regulating ALL cell survival. Here, we found that the level of Cyr61 was increased in the plasma and bone marrow (BM) from ALL patients compared with samples from normal control patients. Furthermore, we observed that Cyr61 could effectively stimulate Jurkat (T ALL cell lines), Nalm-6 (B ALL cell lines), and primary ALL cell survival. Mechanistically, we showed that Cyr61 stimulated ALL cell survival via the AKT/NF-κB signaling pathways and the consequent up-regulation of Bcl-2. Taken together, our study is the first to reveal that Cyr61 is elevated in ALL and promotes cell survival through the AKT/NF-κB pathway by up-regulating Bcl-2. Our findings suggest that Cyr61 plays an important role in the pathogenesis of ALL.

Acute lymphoblastic leukemia (ALL) is characterized by the monoclonal and/or oligoclonal proliferation of hematopoietic precursor cells in the bone marrow (BM). Although it has long been recognized that genetic abnormalities are critical for the development of ALL, increasing evidence suggests that BM stromal cell-derived soluble factors contribute to the pathogenesis of ALL^1^,^2^,^3^,^4^,^5^.

BM stromal cell-derived soluble factors mainly include extracellular matrix molecules, cytokines and chemokines. BM stromal cell-derived soluble factors and BM stromal cells (mesenchymal stem cells, osteoblasts, fibroblasts, adipocytes) form a highly organized three-dimensional scaffold and provide a permissive environment for leukemogenesis and progression^4^,^5^,^6^. Previous studies have shown that stromal-cell-derived factor 1 (SDF-1), interleukin (IL)-3, IL-7, IL-8, CCL2, and stem cell factor (SCF), alone or in different combinations, were shown to promote the survival and (or) the proliferation of ALL cells, although the degree of stimulation is heterogeneous between patient samples and between growth factors^7^,^8^,^9^,^10^,^11^. Levels of basic fibroblast growth factor (bFGF), vascular endothelial factor (VEGF), IL-8 and CCL2 are increased in the BM plasma from ALL patients, and these growth factors can enhance angiogenesis and increase the adhesion of ALL cells to BM stromal cells; in some cases, these growth factors may contribute to the development of ALL^12^,^13^. Together, these results suggest that BM stromal cell-derived soluble factors play important roles in the pathogenesis of ALL. Thus, studies related to BM stromal cell-derived soluble factors would provide a better understanding of the pathogenesis of ALL and facilitate the design of new treatments.

Cyr61/CCN1 is a secreted extracellular matrix (ECM) protein, which is important for cell proliferation, survival, adhesion, migration and differentiation^14^. As a secreted protein, Cyr61 binds to integrins and promotes the development of tumors^14^,^15^,^16^. Cyr61 was originally identified as a growth factor-inducible immediate early gene. It is transcriptionally activated within minutes of stimulation by a variety of factors, including growth factors, cytokines, vitamin D3, cortisol and G-protein coupled receptor (GPCR) agonists^17^,^18^,^19^,^20^. Endothelial and epithelial cells, mesangial cells, mesenchymal cells, smooth muscle cells, cardiomyocytes, osteoblasts, trophoblasts and fibroblast-like synoviocytes have been identified as sources of Cyr61^20^,^21^,^22^,^23^. Recent studies have shown that stromal cells are the major source of Cyr61 in bone marrow^24^,^25^. Notably, Cyr61 is involved in stroma-induced chemo-resistance in acute myeloid leukemia (AML), and the inhibition of Cyr61 could block AML cell growth^24^,^25^. However, whether Cyr61 is involved in the pathogenesis of ALL has not yet been explored.

In this study, we explored the level of Cyr61 in ALL patients and investigated the possible role of Cyr61 in ALL cell survival. We found that Cyr61 is increased in the plasma and the BM from ALL patients. Furthermore, we observed that Cyr61 could effectively promote ALL cell survival through the AKT/NF-κB pathway by up-regulating Bcl-2 and that this effect was abrogated using neutralizing antibodies against human Cyr61. To our knowledge, this study is the first to reveal that the level of Cyr61 is increased in ALL patients and that Cyr61 plays a critical role in ALL cell survival. Our findings suggest that Cyr61 plays an important role in the development of ALL.

## Results

### The level of Cyr61 is increased in the plasma and bone marrow (BM) from ALL patients

**Figure 1:**
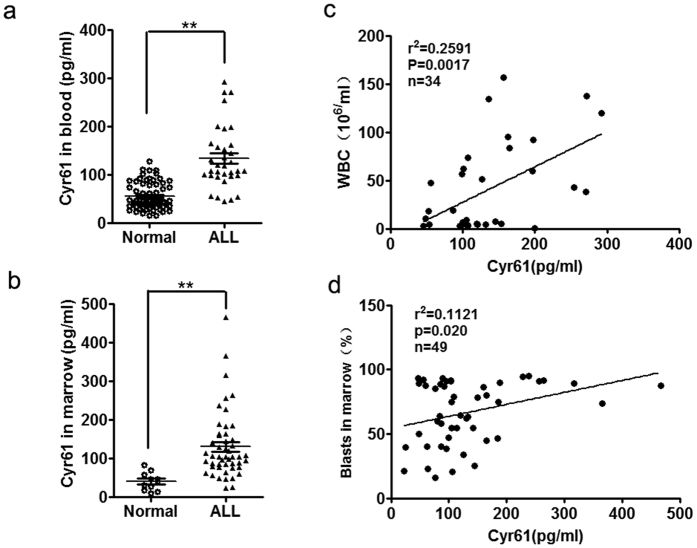
The level of Cyr61 is increased in the plasma and bone marrow (BM) from ALL patients. (**a**) The levels of Cyr61 in the plasma from newly diagnosed without any treatment ALL patients (n = 34) and the normal plasma from age-matched healthy individuals (n = 66) were detected by ELISA. (**b**) The levels of Cyr61 in the BM from newly diagnosed without any treatment ALL patients (n = 49) and the normal BM from age-matched healthy transplant donors (n = 11) were detected by ELISA. (**c**) The relationship of Cyr61 concentrations in the plasma from ALL patients with the number of white blood cells (WBCs) in the peripheral blood was analyzed (n = 33). (**d**) The relationship of Cyr61 concentrations in the BM from ALL patients with the percent of blasts in the BM was analyzed (n = 49). **P < *0*.*01.*

Numerous studies have demonstrated that Cyr61 is an important ECM protein that plays a key role in the pathogenesis of tumors^26^,^27^,^28^,^29^,^30^,^31^; recent studies have shown that Cyr61 is involved in stroma-induced chemo-resistance in acute myeloid leukemia (AML)^24^ and that inhibiting Cyr61 could block AML cell growth^24^,^25^. To explore the role of Cyr61 in the pathogenesis of ALL, we examined Cyr61 concentrations in the plasma and BM from newly diagnosed without any treatment ALL patients (see Supplementary Table S1 and Supplementary Table S2). The results showed that the levels of Cyr61 were increased in the plasma and BM from ALL patients compared with the plasma and BM derived from healthy controls (Fig. 1a, p < 0.0001, **p < 0.01, Student’s t-test; Fig. 1b, p = 0.0012, **p < 0.01, Student’s t-test), and the levels of Cyr61 were not different in the plasma and BM from T-lymphoblastic leukemia (T-ALL) and B-lymphoblastic leukemia (B-ALL) patients (Data not shown). We analyzed the role of Cyr61 in the diagnosis of leukemia and found that the level of Cyr61 in the plasma was significantly related to the number of white blood cells (WBCs), and the level of Cyr61 in the BM was significantly related to the percentage of blasts (Fig. 1c,d). Taken together, these data showed that the level of Cyr61 is increased in ALL patient samples, suggesting that Cyr61 could play a key role in the pathogenesis of ALL.

### Cyr61 effectively stimulated ALL cell survival

**Figure 2:**
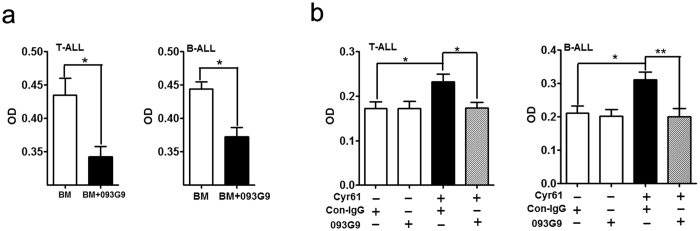
Cyr61 effectively stimulated primary ALL cell survival. (**a**) 3 primary T-ALL cells and 3 primary B-ALL cells (see Supplementary Table S3: P1-P6) were collected and incubated with BM supernatants (Cyr61 concentration is 185 pg/ml) with or without preincubation with anti-human Cyr61 monoclonal antibody (093G9) (1000 pg/ml), and after incubation for 72 h, cell survival was measured using the CCK8 kit. Murine isotype-matched antibody (con-IgG) was used as a control. (**b**) Left panel: Primary T-ALL cell survival was stimulated with 100 ng/ml Cyr61, but Cyr61-stimulated ALL cell survival was inhibited by the addition of the anti-human Cyr61 monoclonal antibody (093G9) (500 ng/ml). Right panel: Primary B-ALL cell survival was stimulated with 1000 ng/ml Cyr61, but Cyr61-stimulated ALL cell survival was inhibited by the addition of the anti-human Cyr61 monoclonal antibody (093G9) (5000 ng/ml). Data represent the mean ± SEM of at least 3 independent experiments. *P < *0*.05, **P < *0*.*01*.

In earlier studies, Cyr61 promoted cell growth in many human tumors^26^,^27^,^29^,^32^,^33^; however, it inhibited the growth of lung cancer cells and liver cancer cell lines^34^,^35^,^36^. To explore whether increased Cyr61 in ALL BM could regulate ALL cell survival, 3 primary T-ALL cells and 3 primary B-ALL cells (see Supplementary Table S3: P1-P6) were cultured in BM supernatants from ALL patients newly diagnosed without any treatment (see Supplementary Table S3: P1-P6), and the results showed that the anti-Cyr61 monoclonal antibody 093G9 could decrease the survival of primary ALL cells cultured in BM supernatants (Fig. 2a, T-ALL p = 0.0349, *p < 0.05, B-ALL p = 0.0151, *p < 0.05, Student’s t-test). Next, 4 primary T-ALL cells and 5 primary B-ALL cells (see Supplementary Table S3: P7-P15) were collected and exposed to recombinant human Cyr61; the results showed that recombinant human Cyr61 could also enhance primary ALL cell survival, and the anti-Cyr61 monoclonal antibody 093G9 could block this effect of Cyr61 on primary ALL cells (Fig. 2b, T-ALL p = 0.0035, *p < 0.05, B-ALL p = 0.0098, **p < 0.01, ANOVA with Student-Newman-Keuls tests). These results showed that Cyr61 could stimulate primary ALL cell survival.

**Figure 3:**
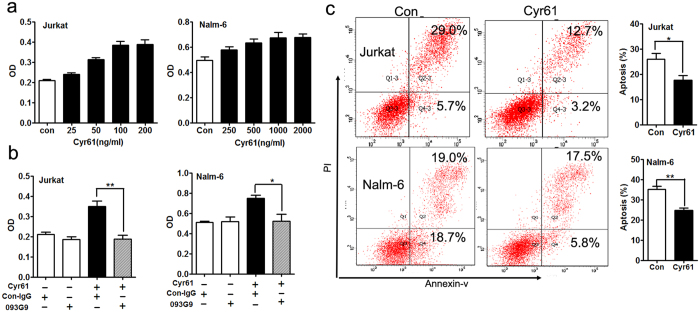
Cyr61 effectively decreased ALL cell apoptosis. (**a**) Left panel: Jurkat cells were stimulated with exogenous recombinant human Cyr61 (25, 50, 100, 200 ng/ml), and after incubation for 72 h, cell survival was measured using the CCK8 kit. Right panel: Nalm-6 cells were stimulated with exogenous recombinant human Cyr61 (250, 500, 1000, 2000 ng/ml), and after incubation for 72 h, cell survival was measured using the CCK8 kit. (**b**) Left panel: Jurkat survival induced by Cyr61 was inhibited by the addition of an anti-human Cyr61 monoclonal antibody (093G9) (500 ng/ml). Murine isotype-matched antibody (con-IgG) was used as a control. Right panel: Nalm-6 survival induced by Cyr61 was inhibited by the addition of an anti-human Cyr61 monoclonal antibody (093G9) (5000 ng/ml). Murine isotype-matched antibody (con-IgG) was used as a control. (**d**) Jurkat and Nalm-6 cells were starved in serum deprivation culture media with or without exogenous recombinant human Cyr61 (100 ng/ml for Jurkat; 1000 ng/ml for Nalm-6) for 48 h, the percentages of apoptotic Jurkat and Nalm-6 (FITC-positive) cells were determined by flow cytometric analysis. Data represent the mean ± SEM of at least 3 independent experiments. *P < *0*.05, **P < *0*.*01*.

To explore the mechanism of Cyr61-stimulated ALL cell survival, T ALL cell lines (Jurkat) and B ALL cell lines (Nalm-6) were exposed to Cyr61 in a suspension culture, and its effects on survival were examined. As shown in Fig. 3a, the survival of Jurkat and Nalm-6 cells were markedly increased in a dose-dependent manner when exposed to Cyr61. We then treated Jurkat and Nalm-6 cells with the anti-Cyr61 monoclonal antibody 093G9 to block Cyr61 function, and the results showed that exposure to 093G9 significantly decreased the effect of Cyr61 on Jurkat and Nalm-6 cell survival (Fig. 3b, Jurkat p = 0.0059, **p < 0.01, Nalm-6 p = 0.0424, *p < 0.05, ANOVA with Student-Newman-Keuls tests). The up-regulation of ALL cell survival by Cyr61 could be due to enhanced cell division and/or reduced cell apoptosis. The CFSE assay showed that Cyr61 had little effect on ALL cell division (see Supplementary Figure S1). Next, we investigated whether Cyr61 could protect ALL cells from apoptosis. Exogenous Cyr61 was added to cultures of starved Jurkat and Nalm-6 cells, and the apoptosis of the Jurkat and Nalm-6 cells was assayed using annexin V-FITC and propidium iodide staining. The apoptosis of the Jurkat and Nalm-6 cells induced by serum deprivation was significantly decreased in the presence of exogenous Cyr61 (Fig. 3c, Jurkat p = 0.0208, *p < 0.05, Nalm-6 p = 0.0046, **p < 0.01, Student’s t-test). These results showed that Cyr61 effectively stimulated ALL cell survival by decreasing cell apoptosis.

### Cyr61 upregulated the Bcl-2 level in ALL cells

**Figure 4:**
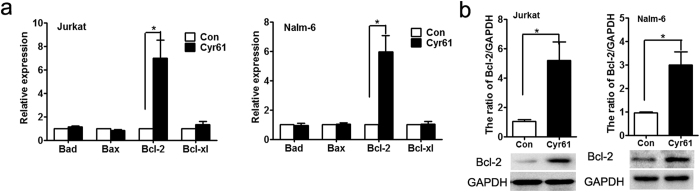
Cyr61 upregulated Bcl-2 levels in ALL cells. (**a**) Left panel: Bcl-2, Bcl-xL, Bax and Bad mRNA expression in Jurkat cells stimulated by 100 ng/ml Cyr61 for 8 h was detected by real-time PCR. Right panel: Bcl-2, Bcl-xL, Bax and Bad mRNA expression in Nalm-6 cells stimulated by 1000 ng/ml Cyr61 for 8 h was detected by real-time PCR. (**b**) Left panel: The protein levels of Bcl-2 in Jurkat cells stimulated by 100 ng/ml Cyr61 for 48 h were detected by western blotting. Right panel: The protein level of Bcl-2 in Nalm-6 cells stimulated by 1000 ng/ml Cyr61 for 48 h was detected by western blotting. The band intensity of Bcl-2 was quantified by densitometry and normalized to GAPDH. Data represent the mean ± SEM of at least 3 independent experiments. *P < *0*.05, **P < *0*.*01*.

Bcl-2 family molecules are important regulators of cell survival and cell death, thus we used real-time PCR to analyze the expression profiles of Bcl-2, Bcl-xL, Bax and Bad in Cyr61-induced ALL cells (Jurkat and Nalm-6). The results showed that in Cyr61-induced Jurkat and Nalm-6 cells, the expression levels of Bcl-2 mRNA were increased significantly (Jurkat p = 0.0185, *p < 0.05, Nalm-6 p = 0.0109, *p < 0.05, Student’s t-test), while the expression levels of Bcl-xL, Bax and Bad did not change (Fig. 4a). Using western blotting, we confirmed that the Bcl-2 protein level was also significantly increased in Cyr61-induced ALL cells (Fig. 4b, Jurkat p = 0.0309, *p < 0.05, Nalm-6 p = 0.0214, *p < 0.05, Student’s t-test). Together, these results suggest that the ALL survival is positively regulated by Cyr61, likely through up-regulating Bcl-2.

### Cyr61-induced ALL cell survival depends on the AKT/NF-κB signaling pathway

**Figure 5:**
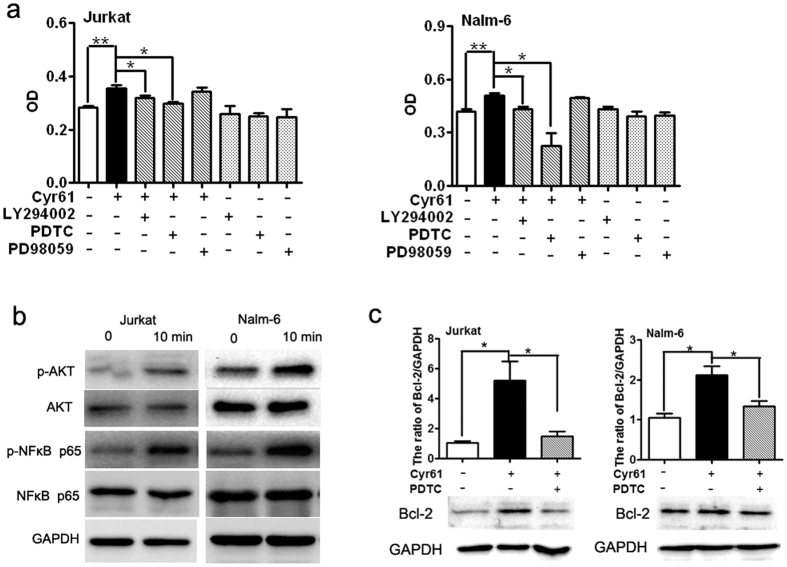
Signaling pathways involved in Cyr61-induced ALL cell survival. (**a**) The effect of the inhibitors of signaling pathways on Cyr61-induced ALL cell survival. Left panel: Jurkat cells were treated with 20 μM LY2940024, 4 μM PDTC or 10 μM SB203580 in combination with Cyr61 (100 ng/ml) (shadow bars) for 72 h, and Jurkat cell survival was evaluated using the CCK8 kit. Right panel: Nalm-6 cells were treated with 20 μM LY2940024, 4 μM PDTC or 10 μM SB203580 in combination with Cyr61 (1000 ng/ml) (shadow bars) for 72 h, and Nalm-6 survival was evaluated using the CCK8 kit. Control (open bar); Cyr61 (no inhibitors, black bar). (**b**) The phosphorylation of AKT and NF-κB was detected by western blotting. Lane 1: unstimulated FLS, lane 2: stimulated with Cyr61 (100 ng/ml for Jurkat; 1000 ng/ml for Nalm-6) for 10 min. (**c**) Left panel: The protein levels of Bcl-2 in Jurkat cells stimulated by 100 ng/ml Cyr61 for 48 h, with or without preincubation with PDTC, were detected by western blotting. Right panel: The protein levels of Bcl-2 in Nalm-6 cells stimulated by 1000 ng/ml Cyr61 for 48 h, with or without preincubation with PDTC, were detected by western blotting. The band intensity of Bcl-2 was quantified by densitometry and normalized to GAPDH. Data represent the mean ± SEM of at least 3 independent experiments. *P < *0*.05, **P < *0*.*01*.

To investigate the mechanism of Cyr61-induced ALL cell survival, Jurkat and Nalm-6 cells were further studied. Previous studies have shown that NF-κB, ERK1/2 and PI3K-AKT are activated downstream of Cyr61 and are associated with proliferation in cancer cells^15^,^37^,^38^,^39^; thus, we probed downstream signaling pathway(s) using known inhibitors of several pathways, including Ly294002 (an inhibitor of the PI3K/AKT pathway), PDTC (an inhibitor of NF-κB activation), and PD98059 (an inhibitor of ERK1/2). The results showed that Cyr61-induced ALL cell (Jurkat and Nalm-6) survival was markedly decreased in the presence of the PI3K/AKT and NF-κB inhibitors. In contrast, the inhibition of ERK1/2 activity had no effect on Cyr61-induced ALL cell survival (Fig. 5a). Further analysis showed that Cyr61 treatment led to a dramatic increase in the phosphorylation of the AKT and NF-κB p65 subunit in Jurkat and Nalm-6 cells (Fig. 5b). Next, we treated cells with the NF-κB inhibitor PDTC to determine whether NF-κB is associated with the Cyr61-mediated regulation of Bcl-2. Our results indicated that treatment with PDTC blocked Cyr61’s up-regulation of Bcl-2 expression in ALL cells (Fig. 5c, Jurkat p = 0.0412, *p < 0.05, Nalm-6 p = 0.0427, *p < 0.05, ANOVA with Student-Newman-Keuls tests). Together, these results suggest that the ALL survival is positively regulated by Cyr61, likely through the AKT/NF-κB pathway by up-regulating Bcl-2.

## Discussion

In this study, we showed that the level of Cyr61 is increased in the plasma and BM from ALL patients. Furthermore, we provide evidence that Cyr61 could effectively promote ALL cell survival. These findings suggested that Cyr61 plays an important role in the development of ALL.

Previous studies have shown that Cyr61 is overexpressed in solid tumors^14^,^15^,^16^. Recent studies have shown that BM stromal cells are the major source of Cyr61^24^,^25^. Our study provides evidence that the level of Cyr61 was increased in the plasma and BM from ALL patients. Furthermore, considering that white blood cell (WBC) count in peripheral blood at diagnosis of ALL is considered to be one of independent risk factors and is used for the risk-group stratification^40^,^41^,^42^,^43^, we evaluated the correlation between the number of WBCs and the level of Cyr61 in peripheral blood from ALL patients. We found that in peripheral blood, the level of Cyr61 was significantly related to the number of WBCs. Importantly, we also found that in the BM from ALL patients, the level of Cyr61 was significantly related to the percentage of blasts, which is important for diagnosing ALL. This finding suggests that the level of Cyr61 is increased in ALL plasma and BM, and Cyr61 may play an important role in the pathogenesis of ALL.

It is well known that ALL is characterized by the abnormal proliferation of hematopoietic precursor cells in the BM. Numerous observations support the fact that Cyr61 could promote the survival and (or) proliferation of tumor cells^26^,^27^,^29^,^32^,^33^, and a recent study showed that inhibiting Cyr61 could block AML cell growth^25^. Our study showed that Cyr61 could promote ALL cell survival by reducing apoptosis; however, Cyr61 has little effect on ALL cell division. In this study, we used recombinant human Cyr61 expressed using an *E. coli* system (PeproTech, Rocky Hill, NJ, USA) for the cell survival assay, and the activity of the protein expressed by *E. coli* was lower than the native protein or that expressed by eukaryotic systems^44^. In addition, Cyr61’s effectiveness may depend on the bone marrow microenvironment, and recombinant human Cyr61 is used in a completely different cell culture condition. Thus, we used higher doses of recombinant human Cyr61 than the amount of endogenous Cyr61 detected in the patients’ bone marrow to test its effect on cell survival *in vitro* in this study. Moreover, we observed that Jurkat cells (T-ALL cell lines) were more sensitive to Cyr61-induced survival than Nalm-6 cells (B-ALL cell lines), which indicates that different cells have different sensitivities to Cyr61. We speculate that this difference may be caused by the different expression of Bcl-2 in Cyr61-induced jurkat cells and Cyr61-induced Nalm-6 cells. Together, these findings demonstrate for the first time that Cyr61 decreases ALL cell apoptosis and promotes cell survival. The findings reported here are consistent with our previous results in which Cyr61 decreased the apoptosis of fibroblast-like synoviocytes from rheumatoid arthritis patients^21^. In addition, previous studies have shown that Cyr61 could decrease the apoptosis of tumor cells, resulting in chemotherapy resistance in breast cancer, cervical cancer and acute myeloid leukemia^24^,^45^,^46^. Cyr61 may be one of the causes of drug resistance in ALL. Our previous studies have shown that blocking Cyr61 activity with a specific mAb ameliorated the severity of the disease in rheumatoid arthritis^47^,^48^ and psoriasis^49^. According to the present and previous studies, Cyr61 plays important roles in the survival of ALL cells, and blocking the Cyr61 pathway may be used for ALL treatment.

The regulation of cell survival is determined by the balance of anti-apoptotic and pro-apoptotic factors of Bcl-2 family proteins^50^, thus we evaluated the effect of Cyr61 on the expression of Bcl-2, Bcl-xL, Bax and Bad as a possible mechanism for Cyr61-induced survival. Our findings showed that Cyr61 could up-regulate Bcl-2 levels without affecting Bcl-xL, Bax or Bad. Considering that Bcl-2 acts as an anti-apoptotic factor, our findings suggested that Cyr61 regulates the survival and apoptosis of ALL cells through the Bcl-2 pathway.

It has been shown that AKT/NF-κB and ERK1/2 pathway are activated downstream of Cyr61 and could up-regulate anti-apoptotic Bcl-2^15^,^37^,^38^,^39^,^51^,^52^,^53^,^54^,^55^. Thus, to address the signaling pathway of Cyr61-induced ALL cell survival, we evaluated the profile of the AKT/NF-κB and ERK1/2 pathways. As expected, the AKT/NF-κB pathway contributed to Cyr61-induced ALL cell survival. Recent evidences suggest that, in AML, Cyr61 mediated AML cell growth by the ERK1/2 pathway^25^; however, our findings showed that ERK1/2 did not contribute to Cyr61-induced ALL cell survival. Because early studies have shown that Cyr61 induces NF-κB activation via the PI3K/AKT pathway in breast cancer cells and fibroblast-like synoviocytes^56^,^57^,^58^,^59^, we suggest that Cyr61-induced ALL cell survival depends on the AKT/NF-κB signaling pathway. Next, we treated ALL cells with the NF-κB inhibitor PDTC and found that treatment with PDTC could block Cyr61’s up-regulation of Bcl-2 expression. Together, these results show that Cyr61-induced ALL cell survival involves the activation of the AKT/NF-κB pathway and the consequent up-regulation of the Bcl-2 pathway. Since BM microenvironment contains lots of growth factors^4^,^5^,^6^, and our study about the signaling pathway of Cyr61-induced ALL cell survival is *in vitro* study and absent other elements of the BM microenvironment, the signaling pathway of Cyr61-induced ALL cell survival *in vivo* need further study.

In this study, we explored the level of Cyr61 in the plasma and BM from ALL patients and investigated the possible role of Cyr61 in the development of ALL. We found that the level of Cyr61 is increased in the BM and plasma from ALL patients. Furthermore, we observed that Cyr61 could effectively stimulate ALL cell survival via the AKT/NF-κB signaling pathways with the consequent up-regulation of the Bcl-2 pathway. Our study indicated for the first time that Cyr61 plays an important role in the development of ALL.

## Methods

### Patients and specimens

ALL samples were obtained from the Affiliated Union Hospital of Fujian Medical University. The supernatant samples from BM (n = 48) were obtained after centrifugation of the total BM aspirates of consecutive ALL patients. Normal BM samples (n = 11) were obtained from the age-matched healthy transplant donors included as controls. Plasma samples from peripheral blood (n = 34) were collected after the centrifugation of peripheral blood from newly diagnosed without receiving any treatment ALL patients. Normal peripheral blood samples (n = 66) were obtained from the age-matched healthy individuals included as controls. The clinical characteristics of the ALL patients are shown in Supplementary Table S1 and Supplementary Table S2. For the survival studies of primary ALL cells cultured with BM supernatants, BM samples from newly diagnosed without receiving any treatment ALL patients (see Supplementary Table S3: P1-P6) were centrifuged at 500 g for 10 min, and supernatants were collected and immediately stored at −80 °C until used. The leukemia cell lines Jurkat and Nalm-6 were kindly provided by Dr. Chen (Shanghai Jiao Tong University School of Medicine, Shanghai, China) and were maintained in RPMI 1640 medium (Hyclone, Logan, UT, USA) supplemented with 10% fetal bovine serum (Gibco, Carlsbad, CA, USA), 100 U/ml penicillin and 100 mg/ml streptomycin at 37 °C and 5% CO_2_. For the survival study, primary ALL cell samples from patients with ALL at the time of diagnosis were enriched for mononuclear cells by density centrifugation and cryopreserved; 7 patients with T-ALL and 8 patients with B-ALL were included (see Supplementary Table S3). Cells were carefully thawed in warm RPMI 1640 with 10% FBS and were cultured for research at 37 °C and 5% CO_2_. These studies were performed in accordance with the ethical guidelines under the protocols approved by the Institutional Medical Ethics Review Board of the Affiliated Union Hospital of Fujian Medical University, Fuzhou, China. Informed consent was obtained from all individual participants included in the study.

### ELISA

The concentration of Cyr61 in the serum and BM from ALL patients was determined using a sandwich ELISA (R&D Systems, Minneapolis, MN, USA) according to the manufacturer’s instructions. Three internal quality control serum samples or BM supernatants were tested in each assay to assess inter-assay precision. The calculated overall inter-assay coefficient of variation was 3.6% to 4.9%.

### ALL cell survival and antibody neutralization assays

Approximately 2.5 × 10^4 ^cells/well Jurkat, 5 × 10^4 ^cells/well Nalm-6 and 5 × 10^4 ^cells/well primary ALL cells were plated in 96-well plates with 100 μl RPMI 1640 with 5% FBS and Cyr61 at different concentrations. For the antibody blocking assay, Cyr61 (PeproTech, Rocky Hill, NJ, USA) was preincubated for 1 h with mouse anti-Cyr61 mAb (093G9) [Anti-Cyr61 mAb was kindly provided by Dr. Li (Shanghai Jiao Tong University School of Medicine, Shanghai, China)] before the stimulation of ALL cell survival. An isotype-matched antibody was used as a control. After incubation for 72 h, cell viability was measured by adding 10 μl CCK8 reagent (Promega, Madison, WI, USA) and incubated for 2 h; then, the plates were monitored using a Power Wave XS microplate reader (BIO-TEK) at 450 nm absorbance.

### Apoptosis assay

For apoptosis assays, apoptotic Jurkat and Nalm-6 cells were induced by serum deprivation in the presence or absence of Cyr61 (100 ng/ml for Jurkat and 1000 ng/ml for Nalm-6) for 48 h and were detected using an annexin V-fluorescein isothiocyanate (FITC) apoptosis detection kit (BD Biosciences, San Jose, CA, USA) according to the manufacturer’s instructions. The percentages of apoptotic Jurkat and Nalm-6 (FITC-positive) cells were determined by flow cytometric analysis. Flow cytometry was performed using a FACS Calibur cytometer (BD Biosciences, San Jose, CA, USA) and analyzed using Cellquest software (BD Biosciences, San Jose, CA, USA).

### Real-time PCR analysis

Total RNA was extracted from specimens using a Tripure isolation reagent (Roche Diagnostics, Indianapolis, IN, USA) according to the manufacturer’s instructions. Total RNA (1 μg) was reverse transcribed into first strand cDNA using the RevertAid^TM^ First Strand cDNA Synthesis Kit (Thermo Scientific, Maryland, USA). Briefly, 1 μl of 50 μM oligo(dT)20 and 1 μl of 10 mM dNTPs mix were added to the RNA, and the volume was adjusted to 11 μl using RNase-free water. Messenger RNA (mRNA) was converted to cDNA according to the manufacturer′s instructions. Real-time PCR was performed using SYBR Green Master Mix (Applied Biosystems, Foster City, CA, USA) according to the manufacturer’s instructions. The primers used in this study were as follows: Bcl-2, forward, CTGGTGGGAGCTTGCATCAC; Bcl-2, reverse, ACAGCCTGCAGCTTTGTTTC; Bcl-xl, forward, TCAGGCTGCTTGGG ATAAAGAT; Bcl-xl, reverse, AGAGGCTTCTGGAGGACATTTG; Bax, forward, TGGAGCTGCAGAGGATG ATTG; Bax, reverse, CCAGTTGAAGTTGCCGTCAG A; Bad, forward, TAAGTC GCGAGCCAGGTTTAAC; Bad, reverse, AGACGCGGGCTTTATTAACATTT; GAPDH, forward, CACATGGCCTCCAAGGAGTA; GAPDH, reverse, TGAGGGT CTCTCTCTTCCTCTTGT.

### Probing of the signaling pathways involved in Cyr61-induced ALL cell survival

Special inhibitors of the AKT and NF-κB signaling pathways were purchased from Sigma-Aldrich and used to analyze Cyr61-induced ALL cell survival. Briefly, 4 μM pyrrolidine dithiocarbamate (PDTC; an inhibitor of NF-κB activation) or 20 μM Ly294002 (an inhibitor of the PI3K/AKT pathway) was added to the cell culture, and Cyr61 was added at the same time. After incubation for 3 days, cell viability was measured by adding 10 μl CCK8 reagent (Promega, Madison, WI, USA) for a 2 h incubation, and then the plates were monitored using a Power Wave XS microplate reader (BIO-TEK) at an absorbance of 450 nm.

### Western blot analysis

Protein immune blotting was performed as described previously^21^. In briefly, tissue or cell lysates were separated by SDS–PAGE electrophoresis followed by transferring to PVDF membranes (Millipore Corporation, Bedford, MA, USA) at 100 v for 90 min. The phosphoralation of AKT and NF-κB were analyzed using specific antibodies (Cell Signaling Technology Inc, Beverly, MA, USA). After washing with PBS, the membranes were incubated with HRP-conjugated goat anti-mouse IgG at RT for 1 h followed by washing with PBS. The target proteins were examined with ECL system (Millipore Corporation, Bedford, MA, USA) and visualized with autoradiography film.

### Statistical analysis

The results were presented as the mean ± SEM unless indicated otherwise. Statistical analyses were performed using the SPSS software version 13.0 (SPSS Inc., Chi cago, IL, USA). The significance of difference between groups was assessed by the Student’s *t* test for single comparisons or by the analysis of variance (ANOVA) with the Student-Newman-Keuls tests for multiple comparisons. A value of *P* < 0.05 was considered as statistical significance.

## Additional Information

**How to cite this article**: Zhu, X. *et al*. Cyr61 participates in the pathogenesis of acute lymphoblastic leukemia by enhancing cellular survival via the AKT/NF-κB signaling pathway. *Sci. Rep.* **6**, 34018; doi: 10.1038/srep34018 (2016).

## Supplementary Material

Supplementary Information
