# Supplementary material for: Cyr61 participates in the pathogenesis of acute lymphoblastic leukemia by enhancing cellular survival via the AKT/NF-κB signaling pathway
Source: Sci Rep. 2016 Oct 11;6:34018. doi: 10.1038/srep34018 (PMC5057070; doi:10.1038/srep34018)
Supplement: Supplementary Information [file srep34018-s1.doc]

**SUPPLEMENTARY INFORMATION**

**Cyr61 participates in the pathogenesis of acute lymphoblastic leukemia by enhancing cellular survival**

**via AKT/NF-κB signaling pathway**

Xianjin Zhu, Yanfang Song, Conglian Wu, Chuxi Pan, Pingxia Lu, Meihua Wang, Peizheng Zheng, Rongfen Huo, Chenqing Zhang, Wanting Li, Yulin Lin, Yinping Cao, Ningli Li

**Supplementary Figure S1. Effects of Cyr61 on ALL cell division.** (A) Up: Jurkat cells were treated with exogenous recombinant human Cyr61 (100 ng/ml), after incubation for 72h, cell division was measured by flow cytometric analysis. Down: Nalm-6 cells were treated with exogenous recombinant human Cyr61 (1000ng/ml), after incubation for 72h, cell division was measured by flow cytometric analysis. Different color peaks represent FLS from different passages labeled with carboxyfluorescein succinimidyl ester (CFSE).


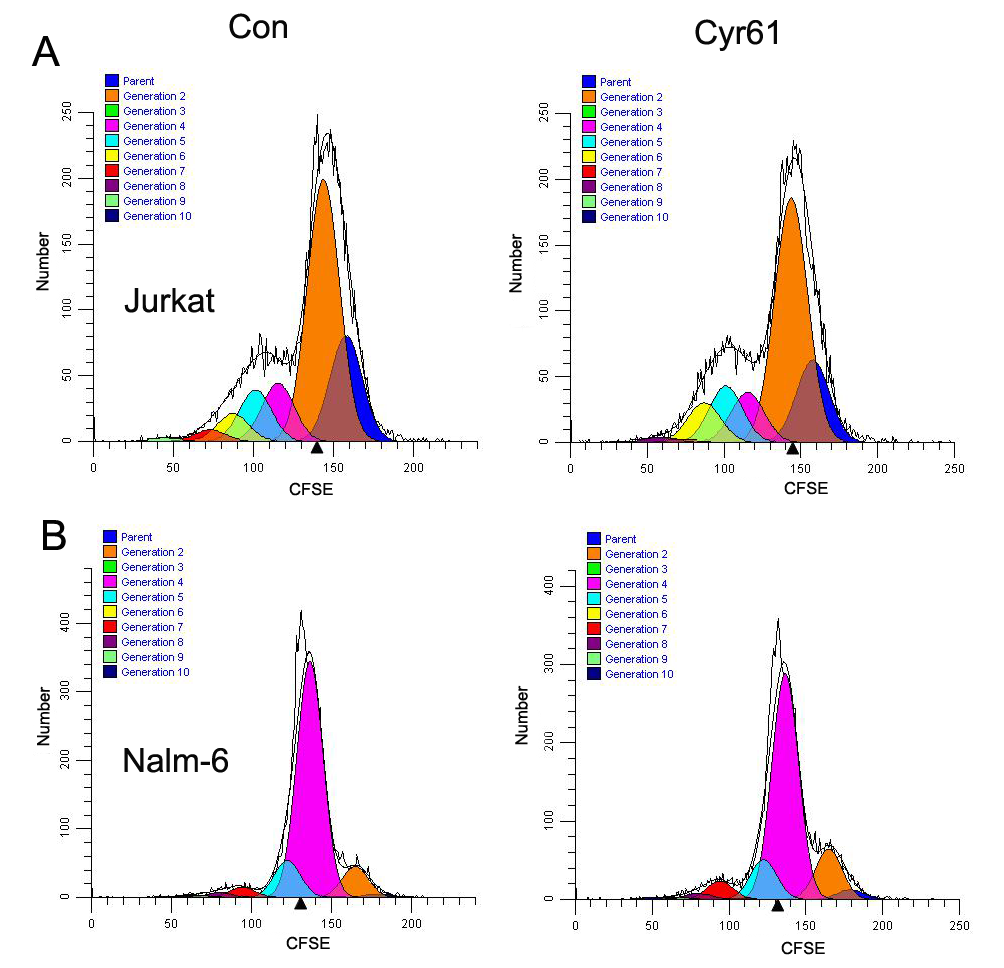


**Supplementary Table S1. Patient Characteristics for the detection of Cyr61 concentrations in BM from ALL patients**

|  | ALL (n=48) | Control (n=11) |
| --- | --- | --- |
| Sex |  |  |
| Male | 27 | 6 |
| Female | 21 | 5 |
| Age (years) | 25.417±19.67 | 25.18±15.34 |
| Diagnosis |  |  |
| T-ALL | 6 |  |
| B-ALL | 42 |  |

**Supplementary Table S2. Patient Characteristics for the detection of Cyr61 concentrations in plasma from ALL patients**

|  | ALL (n=34) | Control (n=66) |
| --- | --- | --- |
| Sex |  |  |
| Male | 22 | 38 |
| Female | 12 | 28 |
| Age (years) | 23.88±21.54 | 27.62±13.50 |
| Diagnosis |  |  |
| T-ALL | 11 |  |
| B-ALL | 23 |  |
|  |  |  |

**Supplementary Table S3. Patient Characteristics for the Survival Studies of BM Derived Cells**

|  | Age | Gender | BM blast cells (%) | Diagnosis |
| --- | --- | --- | --- | --- |
| P1 | 7 | 2 | 89.5 | B-ALL |
| P2 | 15 | 1 | 76.8 | B-ALL |
| P3 | 22 | 1 | 62.3 | B-ALL |
| P4 | 25 | 2 | 91.2 | T-ALL |
| P5 | 46 | 1 | 77.2 | T-ALL |
| P6 | 56 | 1 | 85.3 | T-ALL |
| P7 | 3 | 1 | 86.5 | B-ALL |
| P8 | 56 | 1 | 87 | B-ALL |
| P9 | 4 | 1 | 93 | B-ALL |
| P10 | 31 | 1 | 89.5 | B-ALL |
| P11 | 58 | 2 | 84.2 | B-ALL |
| P12 | 14 | 2 | 89.5 | T-ALL |
| P13 | 5 | 2 | 85.5 | T-ALL |
| P14 | 7 | 1 | 79.2 | T-ALL |
| P15 | 15 | 2 | 72.8 | T-ALL |
